# A Reactive Synchronized Motion Controller for Dual-Arm Cooperation with Closed-Chain Constraints

**DOI:** 10.3390/biomimetics11050298

**Published:** 2026-04-24

**Authors:** Fengjia Ju, Zijian Wang, Mingda Ge, Hongzhe Jin, Jie Zhao

**Affiliations:** School of Mechatronics Engineering, Harbin Institute of Technology, Harbin 150001, China; 20b908029@stu.hit.edu.cn (F.J.); 24b308009@stu.hit.edu.cn (Z.W.); gemingda@outlook.com (M.G.); jzhao@hit.edu.cn (J.Z.)

**Keywords:** synchronized motion control, closed-chain constraints, dual arms, quadratic programming

## Abstract

When a rigid object is manipulated by dual arms to form a closed chain, the dual-arm motion must satisfy closed-chain constraints. Although synchronized motion can be achieved by strictly tracking predefined global trajectories, the presence of dynamic obstacles necessitates reactive local planning. However, existing local planning methods designed for single-arm manipulators cannot guarantee synchronization between dual arms. To address this limitation, we propose a dual-arm reactive synchronized motion controller (SMC) by incorporating closed-chain constraints on dual-arm slack velocities based on spherical geometric velocity constraints, and by implementing a flexible master-slave arm switching strategy. As a result, the proposed controller achieves synchronized dual-arm control while preserving excellent motion performance, including manipulability enhancement, obstacle avoidance, and compliance with joint angle and velocity constraints. Simulations and experiments on a humanoid upper-body robot validate the effectiveness of the proposed approach.

## 1. Introduction

Dual-arm robots offer advantages over single-arm systems, including higher load capacity and improved operational efficiency. During dual-arm collaboration, dual arms may perform independent tasks, such as pouring water into a cup [1], or may jointly manipulate non-rigid objects, such as folding clothes [2]. In these scenarios, closed-chain constraints between dual arms are not required, and each arm can be controlled independently. However, when dual arms manipulate a rigid object—such as a steering wheel [3] or a box—unsynchronized motions that violate closed-chain constraints can generate internal forces between the arms and object, potentially causing damage. Therefore, closed-chain constraints must be considered when handling rigid objects with dual arms.

The global trajectories of the dual-arm end-effectors satisfying closed-chain constraints can be obtained by interpolating the center of the dual-arm end-effectors and subsequently leveraging the relative poses between the end-effectors and the center. In addition, sampling-based methods [4,5], such as PRM, can incorporate global information to achieve collision-free path planning with guarantees on optimality. By enforcing closed-chain constraints on both position and orientation during the sampling process, a fully feasible roadmap [6] can be constructed to achieve coordinated manipulation of dual-arm or multi-arm systems through path search and inverse kinematics [7,8].

**Figure 1:**
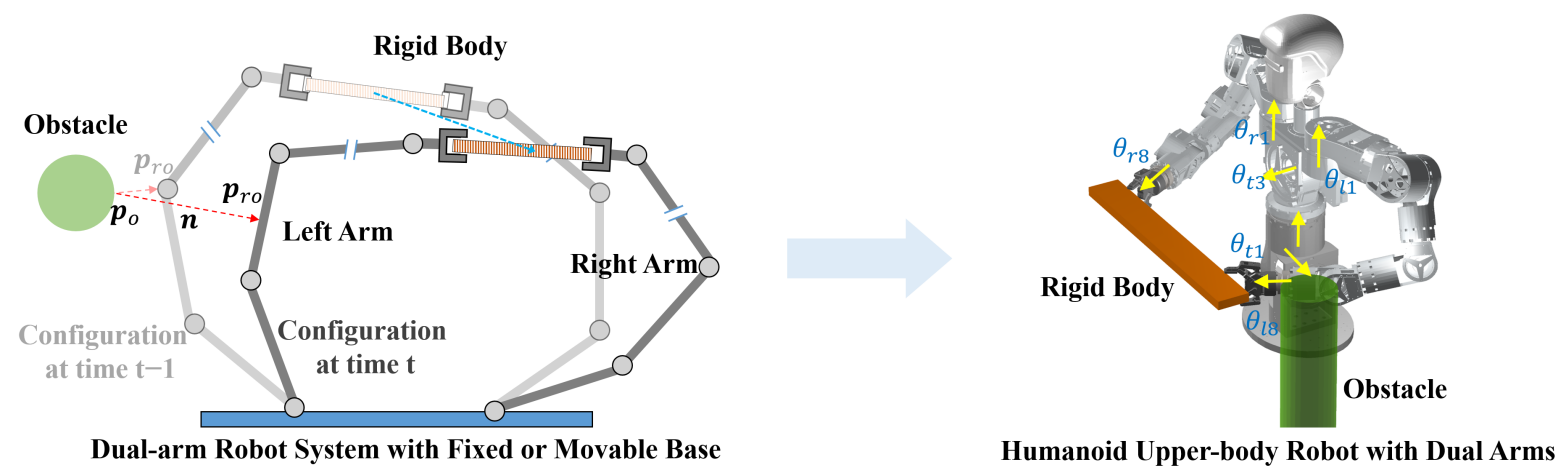
Synchronization motion in the dual-arm system with closed-chain constraints.

However, when a robot operates in environments with dynamic obstacles as shown in Figure 1, trajectories generated by a global planner may become infeasible, necessitating the involvement of a local planner [9,10]. A local planner leverages real-time perception to compute short-horizon, collision-avoidance trajectories. Although global optimality cannot be guaranteed, it enables rapid responsiveness. Reactive approaches [7,8] also fall under local planning methods and offer high computational efficiency. Instead of explicitly generating a planned path, reactive methods directly output control commands as inverse kinematic solutions for the manipulators. Hence, such reactive local planners can also be regarded as local controllers. In order to further improve the tracking ability of the control method in disturbed environments, Refs. [11,12] proposed state filters and multilayer neuroadaptive reinforcement learning methods, respectively. However, reactive adjustments made by local controllers cannot inherently ensure the satisfaction of closed-chain constraints.

Therefore, we aim to develop a local controller capable of maintaining synchronized dual-arm motion. NEO [13] is a velocity-level local controller based on quadratic programming, which considers obstacles, joint limits, and manipulability. By introducing slack velocities, NEO allows the arms to deviate from the desired velocity, and the resulting offsets are exploited to enhance the motion performance. Based on this reactive control framework, we design a motion optimization approach that enforces closed-chain constraints at the velocity level. The slack velocities of dual arms are constrained by the spherical geometric velocity constraint. This constraint is incorporated into the original equality constraints of the velocity-level forward-kinematics in the QP formulation, ensuring that the slack velocities of dual arms satisfy closed-chain constraints.

The main contributions of this work are as follows:

- A reactive synchronized dual-arm motion controller is proposed, enabling point-to-point synchronized inverse kinematics over large motion ranges without trajectory interpolation.
- Based on spherical geometric velocity constraints, equality constraints are established for the synchronized desired velocities and the synchronized slack velocities.
- A master-slave arm selection and switching strategy is designed to enhance manipulability and obstacle avoidance while maintaining closed-chain constraints.

## 2. Related Work

Extensive research has been conducted on synchronized dual-arm motion. Depending on whether force/torque information is incorporated into the control strategy, existing approaches can be broadly classified into dynamic [14,15,16,17,18] or kinematic [6,19,20,21] motion control methods. Furthermore, based on whether the control commands of dual arms are computed independently, these methods can be further categorized into distributed and centralized architectures.

Dynamic motion control typically involves interactions between the grasped object and the environment, such as dual-arm peg-in-hole assembly [14] or dual-arm mirror polishing [16]. These methods rely on force/torque sensors to measure internal forces induced by closed-chain constraints, as well as external forces arising from interactions with the environment [17]. In distributed dynamic control architectures [18], each arm independently considers its own tracking error and internal force, whereas centralized dynamic control architectures [15,16,17] construct synchronization errors based on tracking errors and internal forces of both arms.

In contrast, kinematic motion control aims to avoid the generation of internal forces and is typically designed for transportation tasks [6,19,20] that do not involve interactions between the grasped object and the environment. As a result, force/torque sensors are not required to measure either internal or external forces. Employing kinematic models avoids the complexity of dynamic control or the cost associated with force/torque sensors, while facilitating the integration of additional motion functionalities, such as manipulability enhancement and obstacle avoidance. Similarly, kinematic motion control approaches can also be categorized into distributed and centralized control architectures. Distributed architectures [19,21] perform tracking control based on the individual tracking error of each arm, whereas centralized architectures [20] integrate the tracking errors of dual arms to construct synchronization errors, thereby improving coordination and preventing internal force generation.

However, the aforementioned approaches lack flexibility in local control. The flexibility in local control is challenging because the adjustments of the two arms for motion flexibility and dynamic obstacle avoidance must remain synchronized, while an appropriate master-slave relationship for satisfying synchronization must also be determined. Most existing studies focus on tracking globally synchronized trajectories, often considering disturbances such as visual noise, but lack investigation of synchronized control in environments with dynamic obstacles [22,23]. Furthermore, a master-slave control scheme is introduced for obstacle avoidance in [19]; however, it relies on a fixed assignment of master and slave arms and lacks a dynamic switching strategy. Enhancing the flexibility of local control significantly improves the system’s capability to operate in unstructured and complex environments. Without requiring computationally expensive global replanning, the low-level controller can react in real time to resolve local collision risks or singularity-induced deadlocks. Such flexibility also allows the manipulators to deviate from the desired global trajectory for obstacle avoidance, which may temporarily sacrifice the absolute tracking accuracy of the end-effectors. However, this is acceptable for tasks that do not require strict tracking of the global trajectory. Therefore, we adopt a kinematic reactive control perspective and implement slack-velocity control for dual arms under closed-chain constraints within a centralized QP-based solver, and design a master–slave switching strategy. This approach ensures synchronization while simultaneously enhancing motion flexibility and safety through slack velocities.

The rest of this paper is organized as follows: Section 3 introduces our humanoid upper-body robot and extends the single-arm-based NEO method for dual arms. Section 4 details the proposed synchronized motion controller, while Section 5 presents the simulation and experimental results. Finally, conclusions are drawn in Section 6.

## 3. Problem Description and Modeling Process

### 3.1. Forward Kinematics of Humanoid Upper-Body Robot

The Denavit–Hartenberg (DH) parameters of the humanoid upper-body robot are shown in Table 1. The humanoid upper-body robot consists of a 3-DOF torso ($\theta_{t}={\left[\theta_{t1},\theta_{t2},\theta_{t3}\right]}^{T}$) and two 8-DOF arms (left arm $\theta_{l}={\left[\theta_{l1},\theta_{l2},\ldots ,\theta_{l8}\right]}^{T}$; right arm $\theta_{r}={\left[\theta_{r1},\theta_{r2},\ldots ,\theta_{r8}\right]}^{T}$).

The forward kinematics of the robot is expressed as

(1) $$T_{b}^{i}=F\left(\theta \right)$$

where $\theta =\left[\theta_{t};\theta_{l};\theta_{r}\right]\in R^{19}$ denotes the joint angle vector, and $T_{b}^{i}\in R^{4\times 4}$ denotes the left or right end-effector pose with respect to the robot base frame, i.e., $T_{b}^{l}$ or $T_{b}^{r}$.

The corresponding velocity-level relationship of the forward kinematics is given by

(2) $$\dot{X}=J\left(\theta \right)\dot{\theta }$$

where J(θ) is formed by stacking the Jacobian matrices [24] of the “Torso–Left Arm” and “Torso–Right Arm” kinematic chains,

(3) $$J\left(\theta \right)=\left[\begin{array}{ccc} J_{t}^{l} & J_{l}^{l} & 0_{6\times 8}\\ J_{t}^{r} & 0_{6\times 8} & J_{r}^{r} \end{array}\right]\in R^{12\times 19}$$

$J_{t}^{l}=\partial F(\theta_{t};\theta_{l})/\partial \theta_{t}\in R^{6\times 3}$, $J_{t}^{r}=\partial F(\theta_{t};\theta_{r})/\partial \theta_{t}\in R^{6\times 3}$, $J_{l}^{l}=\partial F(\theta_{t};\theta_{l})/\partial \theta_{l}\in R^{6\times 8}$, $J_{r}^{r}=\partial F(\theta_{t};\theta_{r})/\partial \theta_{r}\in R^{6\times 8}$, $\dot{X}=\left[{\dot{X}}_{l};{\dot{X}}_{r}\right]\in R^{12}$ is the actual velocity vector of the left and right end-effectors, and $\dot{\theta }\in R^{19}$ is the joint angular velocity vector.

The traditional desired velocity combining the velocity forward feedback and the tracking error feedback for a single arm to track a target pose is computed by

(4) $$v={\dot{X}}_{tgt}+k_{p}\cdot E={\dot{X}}_{tgt}+k_{p}\cdot \left(X_{tgt}-X\right)$$

where $k_{p}$ is a gain; E is the tracking error; $X_{tgt}$ and ${\dot{X}}_{tgt}$ are the pose and velocity of the target, respectively; and $X=\left[X_{l};X_{r}\right]\in R^{12}$ is the current left and right end-effector pose vector in the robot base frame. $X_{l}=\Phi \left(T_{b}^{l}\right),X_{r}=\Phi \left(T_{b}^{r}\right)$, where $\Phi \left(\cdot \right):R^{4\times 4}\mapsto R^{6}$ is a function which converts a homogeneous transformation matrix to a pose vector.

### 3.2. Rebuilding NEO Method to Adapt to Dual Arms

To apply NEO [13] to the humanoid upper-body robot, the stacked Jacobian matrix J(θ) in (3) is incorporated, and the task-space dimension of the QP problem is expanded from 6 for a single arm to 12 for dual arms. We rebuild NEO to adapt to dual arms as follows:

(5) $$\begin{array}{cc} \min_{x}\; & f\left(x\right)=\frac{1}{2}x^{T}Qx+C^{T}x\\ s.t.\; & Jx=v,\\ \; & Ax\leq B,\\ \; & x^{-}\leq x\leq x^{+} \end{array}$$

where

(6) $$x=\left[\begin{array}{c} \dot{\theta }\\ \delta \end{array}\right]\in R^{n+12}$$

(7) $$Q=\left[\begin{array}{ccc} \lambda_{\theta }I_{n\times n} & 0_{6\times 6} & 0_{6\times 6}\\ 0_{6\times n} & \lambda_{\delta_{l}}I_{6\times 6} & 0_{6\times 6}\\ 0_{6\times n} & 0_{6\times 6} & \lambda_{\delta_{r}}I_{6\times 6} \end{array}\right]\in R^{\left(n+12)\times (n+12\right)}$$

(8) $$J=\left[\begin{array}{cc} J(\theta ) & I_{12\times 12} \end{array}\right]\in R^{12\times (n+12)}$$

(9) $$C=\left[\begin{array}{c} -J_{m}\\ 0_{12\times 1} \end{array}\right]\in R^{n+12}$$

(10) $$x^{-,+}=\left[\begin{array}{c} {\dot{\theta }}^{-,+}\\ \delta^{-,+} \end{array}\right]\in R^{n+12}$$

Here, *n* is the number of robot joints, $\delta =\left[\delta_{l};\delta_{r}\right]\in R^{12}$ is the slack velocities of the left and right end-effectors, and $J_{m}\in R^{n}$ is the manipulability gradient calculated by the Jacobian and Hessian matrices [13]. The objective function f(x) is derived from $∥\dot{\theta }-J_{m}{)∥}_{2}^{2}$ [25]. By minimizing f(x), the joint velocities calculated via inverse kinematics are driven toward a direction that enhances manipulability.

The manipulability mm is defined by the sum of the manipulabilities [26] of the left and right kinematic chains,

(11) $$mm=mm_{l}+mm_{r}=\sqrt{\det(\left[J_{t}^{l}J_{l}^{l}0_{6\times 8}\right]{\left[J_{t}^{l}J_{l}^{l}0_{6\times 8}\right]}^{T})}+\sqrt{\det(\left[J_{t}^{r}0_{6\times 8}J_{r}^{r}\right]{\left[J_{t}^{r}0_{6\times 8}J_{r}^{r}\right]}^{T})}$$

where det(·) denotes the determinant of a matrix. Manipulability reflects the motion flexibility of a robot. A higher manipulability indicates greater flexibility, a larger distance from singular configurations, and reduced variations in joint velocities during motion, thereby enhancing the safety of the system.

In the previous version of NEO, namely the Manipulability Motion Controller (MMC) [27], a joint velocity damper is introduced to avoid angle limits. Accordingly, the matrices A and B are designed as

(12) $$A=\left[\begin{array}{cc} I_{n\times n} & 0_{n\times 12} \end{array}\right]\in R^{n\times (n+12)}$$

(13) $$B=\eta {\left[\begin{array}{ccc} \frac{\rho_{1}-\rho_{s}}{\rho_{i}-\rho_{s}} & \ldots & \frac{\rho_{n}-\rho_{s}}{\rho_{i}-\rho_{s}} \end{array}\right]}^{T}\in R^{n}$$

where η is a gain, ρ is the distance to the nearest joint limit, $\rho_{s}$ is the minimum allowable distance at which a joint is permitted to approach its limit, and $\rho_{i}$ is the influence distance at which the velocity damper becomes active.

In NEO [13], to further avoid obstacles, the matrices of the obstacle collision damper, $A_{c}$ and $B_{c}$, are formulated based on the closest distance *d* between the robot and obstacles,

(14) $$A_{c}=\left[\begin{array}{cc} J_{d1} & 0_{1\times (n-k_{1})}\\ \vdots & \vdots \\ J_{dw} & 0_{1\times (n-k_{w})} \end{array}\right]\in R^{w\times n}$$

(15) $$B_{c}=\left[\begin{array}{c} \zeta \frac{d_{1}-d_{s}}{d_{i}-d_{s}}-n_{1}^{T}{\dot{p}}_{1}\\ \vdots \\ \zeta \frac{d_{w}-d_{s}}{d_{i}-d_{s}}-n_{w}^{T}{\dot{p}}_{w} \end{array}\right]\in R^{w}$$

where ζ is a gain, *w* is the number of obstacles, $k_{w}$ denotes the index of the robot link corresponding to the closest point to the *w*-th obstacle, ${\dot{p}}_{w}$ is the *w*-th obstacle’s velocity, $n_{w}$ is the unit vector of closest distance from the *w*-th obstacle to the robot, $J_{dw}=-{n_{w}}^{T}J_{p_{ro}}\in R^{1\times k_{w}}$, $J_{p_{ro}}$ is the Jacobian matrix corresponding to the closest distance point on the robot $p_{ro}$, and $d_{s}$ and $d_{i}$ correspond to $\rho_{s}$ and $\rho_{i}$, respectively. To combine the obstacle collision damper with the joint velocity damper, A and B are reformulated as

(16) $$A=\left[\begin{array}{cc} A_{c} & 0_{w\times 12}\\ I_{n\times n} & 0_{n\times 12} \end{array}\right]\in R^{\left(w+n)\times (n+12\right)}$$

(17) $$B={\left[\begin{array}{cccc} {B_{c}}^{T} & \eta \frac{\rho_{1}-\rho_{s}}{\rho_{i}-\rho_{s}} & \ldots & \eta \frac{\rho_{n}-\rho_{s}}{\rho_{i}-\rho_{s}} \end{array}\right]}^{T}\in R^{w+n}$$

Substituting the optimization variable vector $x=[\dot{\theta };\delta ]$ into the equality constraint Jx=v, the equality constraint (8) in NEO can be equivalently written as

(18) $$v-\delta =J\left(\theta \right)\dot{\theta }$$

where δ is introduced into QP as a part of the variables, allowing the end-effectors to deviate from the desired velocity in order to improve overall motion performance. This is reasonable and efficient for transportation scenarios in which the master requirement is accurate and safe arrival at the target pose rather than strictly tracking a global trajectory. However, if no constraints are imposed on $\delta_{l}$ and $\delta_{r}$, the closed-chain constraint may be violated. Therefore, a slack-velocity computation method that explicitly enforces the closed-chain constraint is required, leading to the proposed synchronized motion controller (SMC).

## 4. Synchronized Motion Controller

**Figure 2:**
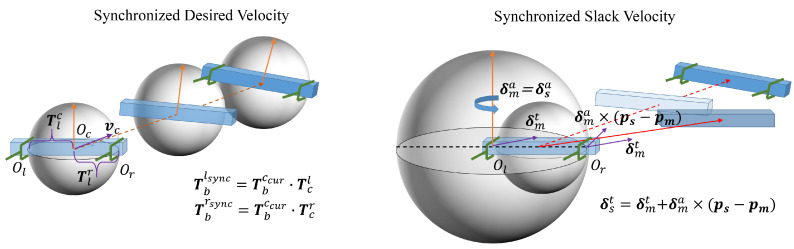
Synchronized desired velocity and synchronized slack velocity based on spherical geometric velocity constraints.

To ensure flexible and synchronized dual-arm motion, the SMC is designed from two perspectives, namely synchronized desired velocity and synchronized slack velocity, as shown in Figure 2. The detailed methodology is described as follows.

### 4.1. Synchronized Desired Velocity

For dual-arm cooperative transportation tasks that form a closed chain, the desired end-effector velocities cannot be computed independently using (4), because the resulting translational velocity is constrained to the straight line connecting the current and target object poses. Pure translational motion satisfies the closed-chain constraint; however, motions involving rotation generally violate this constraint.

When the closed-chain constraint is satisfied, the relative pose between two end-effectors ($O_{l}$ and $O_{r}$) ideally remains equal to the initial relative pose. Accordingly, the line segment connecting the two end-effectors can be regarded as a diameter, and the instantaneous motion space of this segment can be represented by a sphere, as illustrated on the left side of Figure 2.

The proper approach is to first compute the desired velocity of the center point $O_{c}$ of the two end-effectors using (4), and then derive the desired velocities of $O_{l}$ and $O_{r}$ based on their fixed relative poses with respect to the center point.

Specifically, first, the current poses of the left and right end-effectors ($T_{b}^{l_{cur}}$, $T_{b}^{r_{cur}}$) are obtained by (1). Then, exploiting the symmetry between the two end-effector poses, the current pose of the center point $T_{b}^{c_{cur}}$ is computed. Next, the desired velocity of the center point $v_{c}={\left[v_{c}^{x},v_{c}^{y},v_{c}^{z},v_{c}^{ox},v_{c}^{oy},v_{c}^{oz}\right]}^{T}$ is calculated using (4) based on $T_{b}^{c_{cur}}$ and the target pose $T_{b}^{c_{tgt}}$. Finally, the desired velocities of the left and right end-effectors ($v_{l}={\left[v_{l}^{x},v_{l}^{y},v_{l}^{z},v_{l}^{ox},v_{l}^{oy},v_{l}^{oz}\right]}^{T}$, $v_{r}={\left[v_{r}^{x},v_{r}^{y},v_{r}^{z},v_{r}^{ox},v_{r}^{oy},v_{r}^{oz}\right]}^{T}$) are then derived based on the spherical geometric velocity constraints and the respective initial relative pose relationships between each end-effector and the center point, as follows:

(19) $$\left[\begin{array}{c} v_{l}^{ox}\\ v_{l}^{oy}\\ v_{l}^{oz} \end{array}\right]=\left[\begin{array}{c} v_{r}^{ox}\\ v_{r}^{oy}\\ v_{r}^{oz} \end{array}\right]=\left[\begin{array}{c} v_{c}^{ox}\\ v_{c}^{oy}\\ v_{c}^{oz} \end{array}\right]$$

(20) $$\left[\begin{array}{c} v_{l}^{x}\\ v_{l}^{y}\\ v_{l}^{z} \end{array}\right]=\left[\begin{array}{c} v_{c}^{x}\\ v_{c}^{y}\\ v_{c}^{z} \end{array}\right]+\left[\begin{array}{c} v_{c}^{ox}\\ v_{c}^{oy}\\ v_{c}^{oz} \end{array}\right]\times \left(p_{l}-p_{c}\right)$$

(21) $$\left[\begin{array}{c} v_{r}^{x}\\ v_{r}^{y}\\ v_{r}^{z} \end{array}\right]=\left[\begin{array}{c} v_{c}^{x}\\ v_{c}^{y}\\ v_{c}^{z} \end{array}\right]+\left[\begin{array}{c} v_{c}^{ox}\\ v_{c}^{oy}\\ v_{c}^{oz} \end{array}\right]\times \left(p_{r}-p_{c}\right)$$

where $p_{l},p_{r},p_{c}\in R^{3}$ represents the position components in $T_{b}^{l_{sync}}$, $T_{b}^{r_{sync}}$, and $T_{b}^{c_{cur}}$, respectively. $T_{b}^{l_{sync}}=T_{b}^{c_{cur}}\cdot T_{c}^{l}$ and $T_{b}^{r_{sync}}=T_{b}^{c_{cur}}\cdot T_{c}^{r}$ denote the synchronized poses of the left and right end-effectors, respectively, which satisfy the closed-chain constraints with respect to the current center point $T_{b}^{c_{cur}}$. $T_{c}^{l}$ is the initial relative pose between $O_{c}$ and $O_{l}$, while $T_{c}^{r}$ is the initial relative pose between $O_{c}$ and $O_{r}$. × denotes the cross product.

**Remark** **1.** *It should be noted that, if the current actual poses of the left and right end-effectors are directly used in (19)–(21), the resulting synchronized desired velocities may violate the closed-chain constraint. This is because both end-effectors exhibit deviations between their current actual poses and the desired synchronized poses, thereby compromising synchronization. Therefore, when computing the synchronized desired velocities in (19)–(21), the poses of the left and right end-effectors are defined relative to the center point in a manner that preserves the synchronization relationship. In this way, the resulting velocities satisfy the closed-chain constraint. However, due to the discrepancy between the desired and actual poses, an additional compensation term must be introduced into the velocity control commands of the left and right end-effectors to reduce this error and improve synchronization.*

To solve this issue, the velocity compensation terms for the left and right end-effectors ($v_{comp}^{l}$, $v_{comp}^{r}$), respectively, based on their tracking errors are introduced:

(22) $$v_{comp}^{l}=k_{v}\cdot \left(\Phi \left(T_{b}^{l_{sync}}\right)-\Phi \left(T_{b}^{l_{cur}}\right)\right)$$

(23) $$v_{comp}^{r}=k_{v}\cdot \left(\Phi \left(T_{b}^{r_{sync}}\right)-\Phi \left(T_{b}^{r_{cur}}\right)\right)$$

where $k_{v}$ is a gain.

The synchronized desired velocity v used in (5) is composed of $v_{l}$, $v_{r}$, $v_{comp}^{l}$ and $v_{comp}^{r}$,

(24) $$v=[v_{l}+v_{comp}^{l};v_{r}+v_{comp}^{r}]$$

**Figure 3:**
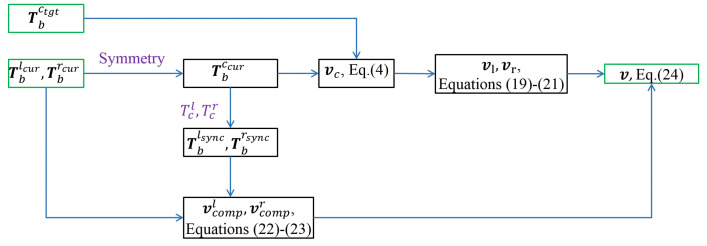
A calculation flowchart of the synchronized desired velocity.

A calculation flowchart of the synchronized desired velocity is shown in Figure 3.

However, an unsynchronized slack velocity δ without constraining the relationship between $\delta_{l}$ and $\delta_{r}$ in (18) can disrupt dual-arm synchronization. Therefore, additional constraints on the slack velocities must be introduced.

### 4.2. Synchronized Slack Velocity

To address the desynchronization arising from independently computed slack velocities, a master-slave strategy is introduced. The slack velocity of the master arm is unconstrained, while the slave arm adapts its slack velocity to that of the master arm to maintain the closed-chain constraint. The purpose of introducing slack velocity is to allow the end-effector to deviate from the desired velocity, facilitating motion optimizations such as manipulability enhancement and obstacle avoidance. However, the constrained slack velocity of the slave arm may reduce its ability to exploit the flexibility provided by slack velocities, forcing it to passively follow the slack motion of the master arm. Therefore, the selection criterion for the master and slave arms should ensure that the arm with weaker capability for flexible adjustment is assigned as the master arm, while the arm with stronger capability serves as the slave arm.

This capability of flexible adjustment is reflected in the robot’s ability to accomplish the master task under different scenarios. The master task is the synchronized target tracking. In obstacle-free environments, the challenge of completing the master task arises from singularity. Higher manipulability indicates that the robot is further from singular configurations. Therefore, the capability of flexible adjustment can be evaluated using manipulability. When manipulability is high, there is less need for slack velocity to adjust the end-effector motion. Even if the slack velocity is constrained by the closed-chain constraint, sufficient flexibility remains to accomplish the master task. Conversely, when manipulability is low, the role of slack velocity becomes more critical.

In environments with obstacles, the challenge of completing the master task is collision. In this case, the capability of flexible adjustment should be evaluated based on the distance to obstacles. Similarly to the manipulability analysis, a larger distance indicates greater flexibility to accomplish the master task. Accordingly, depending on the scenario, the arm with lower manipulability or a closer distance to obstacles is selected as the master arm, while the other serves as the slave arm. In addition, other criteria can be defined for different task scenarios.

The slack motion of the slave arm is modeled as a rotation about the master arm’s end-effector coordinate origin, as shown in the right side of Figure 2. Since the motion is a rotation about a common axis, the angular velocity of any point on the object is identical. Consequently, the slack angular velocities of the master and slave arms are identical and are determined by the slack angular velocity of the master arm. The slack translational velocity of the master arm is computed without enforcing the closed-chain constraint, whereas that of the slave arm is constrained to incorporate both the master arm’s translational component and the velocity induced by rotational motion. The detailed closed-chain constraints at the velocity level are shown as follows:

(25) $$\left[\begin{array}{c} \delta_{m}^{ox}\\ \delta_{m}^{oy}\\ \delta_{m}^{oz} \end{array}\right]=\left[\begin{array}{c} \delta_{s}^{ox}\\ \delta_{s}^{oy}\\ \delta_{s}^{oz} \end{array}\right]$$

(26) $$\left[\begin{array}{c} \delta_{s}^{x}\\ \delta_{s}^{y}\\ \delta_{s}^{z} \end{array}\right]=\left[\begin{array}{c} \delta_{m}^{x}\\ \delta_{m}^{y}\\ \delta_{m}^{z} \end{array}\right]+\left[\begin{array}{c} \delta_{m}^{ox}\\ \delta_{m}^{oy}\\ \delta_{m}^{oz} \end{array}\right]\times \left(p_{s}-p_{m}\right)$$

where $\delta_{m}=\left[\delta_{m}^{t};\delta_{m}^{a}\right]={\left[\delta_{m}^{x},\delta_{m}^{y},\delta_{m}^{z},\delta_{m}^{ox},\delta_{m}^{oy},\delta_{m}^{oz}\right]}^{T}\in R^{6}$ is the slack velocity of the master arm, $\delta_{s}=\left[\delta_{s}^{t};\delta_{s}^{a}\right]={\left[\delta_{s}^{x},\delta_{s}^{y},\delta_{s}^{z},\delta_{s}^{ox},\delta_{s}^{oy},\delta_{s}^{oz}\right]}^{T}\in R^{6}$ is the slack velocity of the slave arm, and $p_{m},p_{s}\in R^{3}$ represents the position vectors of the master and slave arms’ end-effectors. The correspondence between the end-effector positions of the master and slave arms ($p_{m},p_{s}$) and those of the left and right arms ($p_{l},p_{r}$) depends on the designed master-slave switching strategy.

Furthermore, the relationship between the slack velocities of the dual arms in (25) and (26) can be described using a closed-chain constraint matrix $M\in R^{6\times 6}$,

(27) $$\delta_{s}=M\cdot \delta_{m}$$

where

(28) $$M=\left[\begin{array}{cc} I_{3\times 3} & -S(p_{s}-p_{m})\\ 0_{3\times 3} & I_{3\times 3} \end{array}\right]$$

S(·) is the skew-symmetric matrix of a vector. By exploiting the relationship between skew-symmetric matrices and the cross-product operation, the cross-product term in (26) is reformulated using a skew-symmetric matrix in (28) for a more compact representation.

Then, by incorporating the closed-chain constraint matrix M, J in (5) and (8) can be rewritten as

(29) $$J=\left\{\begin{array}{c} \left[\begin{array}{ccc} J(\theta ) & I_{12\times 6} & I_{12\times 6}\\ 0_{6\times 19} & -M & I_{6\times 6} \end{array}\right],\mathrm{if}\;\mathrm{the}\;\mathrm{left}\;\mathrm{arm}\;\mathrm{is}\;\mathrm{the}\;\mathrm{master}\;\mathrm{arm}\\ \left[\begin{array}{ccc} J(\theta ) & I_{12\times 6} & I_{12\times 6}\\ 0_{6\times 19} & I_{6\times 6} & -M \end{array}\right],\mathrm{if}\;\mathrm{the}\;\mathrm{right}\;\mathrm{arm}\;\mathrm{is}\;\mathrm{the}\;\mathrm{master}\;\mathrm{arm} \end{array}\right.$$

The dimension of J is extended from 12×(n+12) to 18×(n+12). Correspondingly, v is extended from a 12-dimensional column vector to an 18-dimensional one by adding $0_{6\times 1}$.

The extended equality constraint (Jx=v) in (5) simultaneously incorporates the constraints in (18) and (27), enabling the dual-arm end-effectors to possess slack velocities while ensuring that these slack velocities satisfy the synchronization constraints.

**Theorem** **1.** *Under bounded perturbations, the tracking error E of the proposed synchronized motion controller (SMC), subject to the constraints in (29), is Uniformly Ultimately Bounded (UUB).*

The proof of Theorem 1 can be found in Appendix A.

### 4.3. Master–Slave Arm Switching Strategy

Building upon the previously introduced master-slave arm switching strategy driven by manipulability and obstacle avoidance, this subsection presents a precise mathematical formulation of the proposed approach.

We define Δ as the difference between the manipulabilities of dual arms or between their minimum distances to obstacles, depending on the operating scenario, and use it as the decision criterion for master-slave arm switching. In obstacle-free environments, $\Delta =mm_{l}-mm_{r}$, and in environments with obstacles, $\Delta =d_{min}^{l}-d_{min}^{r}$. $mm_{l}$, $mm_{r}$, $d_{min}^{l}$ and $d_{min}^{r}$ are respectively the manipulabilities and the minimum distances to the obstacles of the left and right arms.

Without the smoothing process, directly applying a hard switching strategy may lead to abrupt changes in the slack velocities due to the variation in closed-chain constraints in the QP problem before and after switching, thereby causing oscillations in the robot motion. To address this issue, we design a dynamically adjustable gain ϵ based on Δ to smoothly regulate the slack-velocity vector. This parameter is incorporated as a weighted factor in the quadratic term of the QP problem, enabling effective control of the slack-velocity vector.

A straightforward approach to ensure smoothness is to increase ϵ during master-slave switching to suppress the slack velocities, driving them close to zero, and then decrease ϵ after the switching is completed. However, this strategy may introduce excessive constraints and reduce the flexibility provided by the slack velocities. Therefore, a more gradual switching scheme is adopted. As indicated by (25) and (26), under the imposed constraints, the slack angular velocities of the master and slave end-effectors remain identical, whereas differences in their slack translational velocities are induced by the angular slack motion.

Since the slack angular velocity influences the slack linear velocity through (26), we indirectly smooth the slack linear velocity by smoothing the slack angular velocity. Consequently, ϵ is applied exclusively to the slack angular velocity to suppress it toward zero. This approach ensures a smooth transition while preserving the flexible adjustment capabilities provided by the slack velocity. Specifically, it enables the slack velocity to manifest as pure translation during the switching phase, and as a combination of translation and rotation during non-switching periods.

To achieve this effect, ϵ should be designed as a smooth function such that it takes a relatively large value when approaching a switching condition (i.e., Δ→0) and a smaller value when far from switching (i.e., Δ is away from zero).

Based on the above considerations, various forms of ϵ can be adopted. In this work, we select an exponential function due to its simplicity and ease of tuning, defined as

(30) ϵ=a·exp(b·abs(Δ))+1

where $a\in R^{+},b\in R^{-}$. The parameter *a* controls the degree of suppression on the slack angular velocity: a larger *a* results in a larger quadratic weight, leading to stronger suppression and thus smaller slack angular velocity, and vice versa. The parameter *b* determines the range of influence of ϵ with respect to Δ: a larger magnitude of *b* leads to a faster variation of the exponential function, concentrating the effect of ϵ around the switching region; conversely, a smaller magnitude extends its influence, which may undesirably affect the flexibility of slack velocity away from switching. To prevent ϵ from becoming too small—thereby causing excessively large slack velocities and degrading tracking performance—a constant term of 1 is added to ensure that ϵ>1 at all times.

Incorporating ϵ, the weighted factor of the quadratic term in the QP formulation is given as follows:

(31) $$Q=\left[\begin{array}{ccccc} \lambda_{\theta }I_{n\times n} & 0_{3\times 3} & 0_{3\times 3} & 0_{3\times 3} & 0_{3\times 3}\\ 0_{3\times n} & \lambda_{\delta_{l}}^{t}I_{3\times 3} & 0_{3\times 3} & 0_{3\times 3} & 0_{3\times 3}\\ 0_{3\times n} & 0_{3\times 3} & \lambda_{\delta_{l}}^{a}I_{3\times 3} & 0_{3\times 3} & 0_{3\times 3}\\ 0_{3\times n} & 0_{3\times 3} & 0_{3\times 3} & \lambda_{\delta_{r}}^{t}I_{3\times 3} & 0_{3\times 3}\\ 0_{3\times n} & 0_{3\times 3} & 0_{3\times 3} & 0_{3\times 3} & \lambda_{\delta_{r}}^{a}I_{3\times 3} \end{array}\right]$$

where

(32) $$\begin{array}{cc} \; & \lambda_{\delta_{l}}^{t}=k_{\lambda }\cdot \frac{1}{\sum \mathrm{abs}(E_{l})},\;\lambda_{\delta_{r}}^{t}=k_{\lambda }\cdot \frac{1}{\sum \mathrm{abs}(E_{r})},\\ \; & \lambda_{\delta_{l}}^{a}=\epsilon \cdot k_{\lambda }\cdot \frac{1}{\sum \mathrm{abs}(E_{l})},\;\lambda_{\delta_{r}}^{a}=\epsilon \cdot k_{\lambda }\cdot \frac{1}{\sum \mathrm{abs}(E_{r})} \end{array}$$

are the weights associated with the translational and angular slack velocities of dual arms, $E_{l},E_{r}\in R^{6}$ represents the tracking errors of dual arms, $k_{\lambda }$ is the gain, abs(·) is the element-wise absolute value operator, and ∑(·) is the sum of vector elements.

As stated in [13], a higher $\lambda_{\theta }$ reduces the ability to maximize manipulability, whereas an excessively low value may lead to extreme joint velocities. Higher $\lambda_{\delta_{l}}^{t},\lambda_{\delta_{r}}^{t},\lambda_{\delta_{l}}^{a},\lambda_{\delta_{r}}^{a}$ limits the possible additional manipulability achievable, while a lower value leads to the possibility that the slack will cancel out the desired velocity, leaving large tracking errors. Therefore, the weights are combined with tracking errors to dynamically regulate the magnitude of the slack velocities at different motion stages. In the initial phase, when the tracking error is relatively large, motion performance can be improved by increasing the slack velocity. In the final phase, as the error becomes small, the slack velocity is reduced to ensure tracking accuracy. Moreover, to smooth slack velocity during master-slave switching, ϵ is introduced into $\lambda_{\delta_{l}}^{a},\lambda_{\delta_{r}}^{a}$ to suppress the slack angular velocity.

**Remark** **2.** *While this work primarily maximizes manipulability and ensures obstacle avoidance, another crucial aspect in trajectory generation is energy optimization (e.g., minimizing joint movement). In highly constrained dual-arm closed-chain tasks, guaranteeing motion flexibility and collision safety is typically the highest priority, as reaching a singularity or obstacle leads to immediate task failure. Nevertheless, the energy aspect is implicitly partially addressed in our QP formulation. By penalizing the magnitude of the joint velocities (via the weight $\lambda_{\theta }$), the solver naturally suppresses excessive joint excursions, acting as a kinematic proxy for minimizing instantaneous kinetic energy.*

The proposed method is summarized in the following pseudocode in Algorithm 1. For the dual-arm synchronized transportation task considered in this work, the initial and target poses of the two end-effectors are determined by the initial and target poses of the grasped object, as well as a task-specific grasping strategy based on the object’s geometry. In this work, these quantities are assumed to be given, and their determination is not further discussed.
**Algorithm 1:** Synchronized Motion Controller
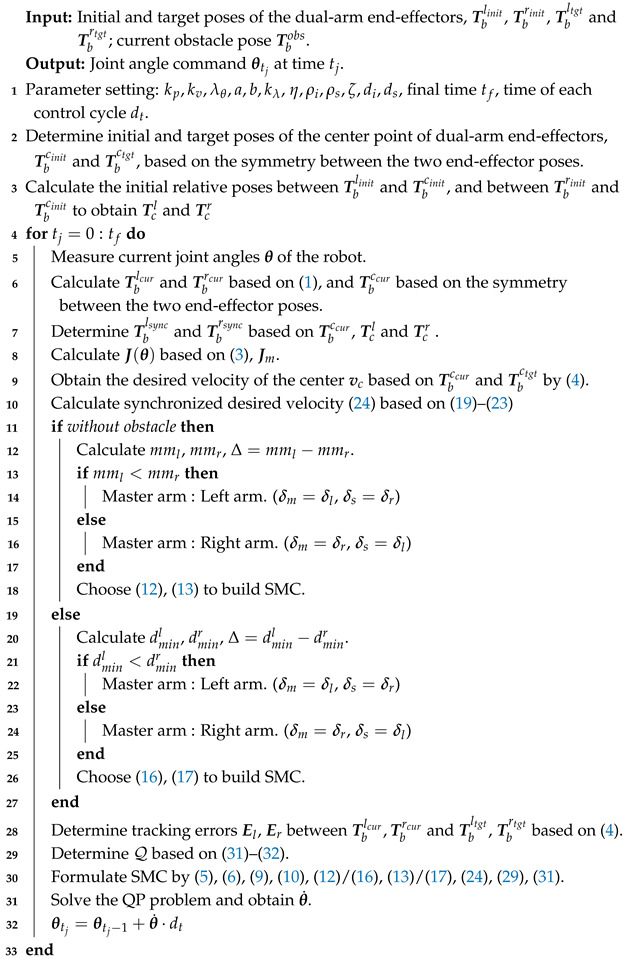

**Remark** **3.** *Our reactive control approach is local in nature and does not guarantee global optimality. When the robot deviates from the global path to avoid obstacles, it may encounter local minima. In practical applications, if a local minimum is reached, the intervention of a global planner is required for re-planning. Specifically, the global planner is triggered to re-calculate a path whenever the SMC tracking error exceeds a predefined threshold. The newly generated global path then provides updated local waypoints at a specific frequency to refresh the target being tracked by the SMC.*

## 5. Simulations and Experiments

### 5.1. Comparison Indexes

The synchronization performance is evaluated using the synchronization position error $E_{s}^{p}$ and synchronization orientation error $E_{s}^{o}$. These errors are computed based on the relative pose vector $E_{rel}={\left[E_{rel}^{p},E_{rel}^{o}\right]}^{T}\in R^{6}$, which is derived from the current poses of the left and right end-effectors, $T_{b}^{l_{cur}}$ and $T_{b}^{r_{cur}}$.

(33) $$E_{rel}=\Phi \left({\left(T_{b}^{l_{cur}}\right)}^{-1}\cdot \left(T_{b}^{r_{cur}}\right)\right)$$

(34) $$E_{s}^{p}=N\left(E_{rel}^{p}\right)-d_{0}$$

(35) $$E_{s}^{o}=N\left(E_{rel}^{o}\right)-\theta_{0}$$

where N(·) denotes the 2-norm of a vector, $d_{0}$ is the initial distance between the two end-effectors, and $\theta_{0}$ is the angle required to rotate from one end-effector’s orientation to the other around an axis in space at the initial moment.

Additionally, the total of the dual-arm tracking position error $E_{t}^{p}$ and the total of the dual-arm tracking orientation error $E_{t}^{o}$ are also considered.

(36) $$E_{t}^{p}=N\left(E_{l}^{p}\right)+N\left(E_{r}^{p}\right)$$

(37) $$E_{t}^{o}=N\left(E_{l}^{o}\right)+N\left(E_{r}^{o}\right)$$

where $E_{l}^{p}$ and $E_{r}^{p}$ are the tracking position error vectors of the left and right arms, and $E_{l}^{o}$ and $E_{r}^{o}$ are the tracking orientation error vectors of the left and right arms.

The parameters of the proposed SMC used in both simulations and experiments are set as follows: $k_{p}=\min\left(1.5\times \left(1-e^{-0.5t_{j}}\right),1.5\right)$ in (4) to smooth the startup according to the time $t_{j}$, $\eta =1,\;\rho_{i}=15^{{}^{\circ}},\;\rho_{s}=5^{{}^{\circ}}$ in (13), $\zeta =1,\;d_{i}=0.35\;m,d_{s}=0.1\;m$ in (17), $k_{v}=1$ in (22), and (23), $\lambda_{\theta }=0.01,\;k_{\lambda }=10,\;a=10,\;b=-100$ in (31), $d_{t}=0.01\;s$.

### 5.2. Simulations

In this section, two sets of comparative simulations are conducted: one without obstacles and one with obstacles. To validate the effectiveness of the proposed method in synchronized motion control, we compare the proposed SMC with two baseline methods: the original QP method [25], which strictly tracks the desired velocity without slack variables; the NEO method [13], which introduces slack velocities but lacks closed-chain constraints; and the proposed SMC. All methods use the synchronized desired velocity in (24) for the dual-arm cooperation tasks. In the task setup, only the initial and target poses are specified, without performing trajectory interpolation.

In addition, to demonstrate the capability of SMC to achieve synchronized tracking without trajectory interpolation, we further compare it with the SyncTC method [20]. The SyncTC method is a centralized synchronized tracking control approach that minimizes joint velocities using quadratic programming. It constructs a coupled error by combining tracking error and synchronization error, and further formulates a zeroing neurodynamic model based on the coupled error, which is incorporated into the QP as a closed-chain constraint. The SyncTC method tracks synchronized trajectories that are precomputed using quintic polynomial interpolation.

#### 5.2.1. Simulation A

**Figure 4:**
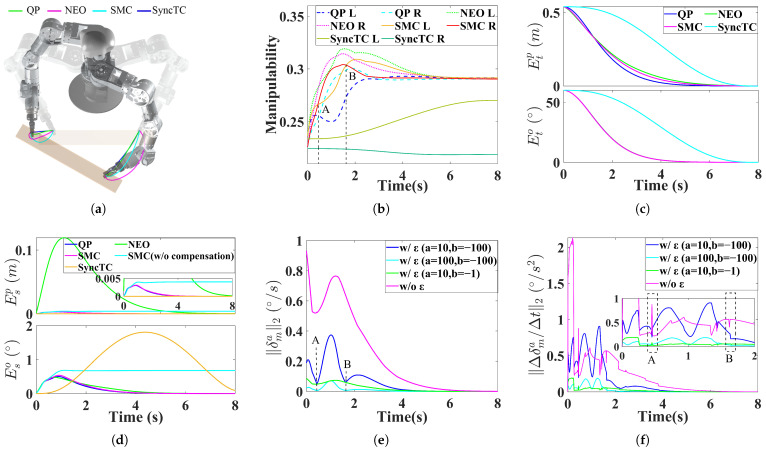
Simulation A. (**a**) Trajectories from a top-down perspective. (**b**) Manipulability. (**c**) Tracking error. (**d**) Synchronization error. (**e**) Slack velocity in SMC. (**f**) Slack acceleration in SMC.

This simulation is conducted in an obstacle-free environment, where manipulability is used as the criterion for master-slave arm switching. The robot motion trajectories generated by the three methods are shown in Figure 4a.

From Figure 4b, the average sums of manipulability of the two arms during motion for the QP, NEO, SMC, and SyncTC methods are 0.571, 0.594, 0.583, and 0.472, respectively. Compared with QP, the NEO and SMC methods, which incorporate slack velocities, improve manipulability by 4.0% and 2.1%, respectively. However, due to the closed-chain constraints imposed in SMC, its manipulability is slightly lower than that of NEO. Compared with SyncTC, which does not include manipulability optimization, SMC improves manipulability by 23.5%.

Figure 4c shows that SMC also achieves high tracking accuracy. At the final time, the sum of the tracking position and orientation errors of the left and right end-effectors under SMC are 0.47 mm and 0.0065°, respectively. However, due to the introduction of slack velocities, the tracking position errors of SMC are larger than those of the QP method (0.15 mm and 0.0066°), and also larger than those of the SyncTC method (0.13 mm and 0.0071°). However, in terms of tracking orientation error, the SMC method outperforms the QP and SyncTC methods.

The synchronization errors shown in Figure 4d indicate that the proposed SMC achieves good synchronization performance, with maximum synchronization position and orientation errors of 3.1 mm and 0.54°, respectively. In contrast, the NEO method without closed-chain constraints results in excessive synchronization position error due to independent slack motions of dual arms, with maximum errors reaching 119.3 mm and 0.48°, respectively. Both the QP method, which strictly tracks synchronized desired velocities, and the SyncTC method, which tracks synchronized trajectories, have smaller synchronization position error, but lack the manipulability benefits provided by slack velocities. Their maximum synchronization position and orientation errors are 3.0 mm and 0.50°, and 0.02 mm and 1.81°, respectively. In terms of synchronization orientation error, the SMC method outperforms the QP and SyncTC methods.

Furthermore, to demonstrate the effect of the compensation velocities $v_{comp}^{l}$ and $v_{comp}^{r}$ introduced in the SMC method, we remove these terms for comparison. Without compensation, the synchronization error increases, reaching up to 4.0 mm and 0.69°.

Figure 4e,f present the slack angular velocity and slack angular acceleration in the SMC method. To illustrate the role of the dynamic gain ϵ during master-slave switching, we consider cases without ϵ and with different parameter settings. As shown in Figure 4b, the SMC method (with a=10,b=−100) performs master-slave switching at points A and B. At these moments, the slack angular velocity exhibits a noticeable decrease, while the slack angular acceleration remains relatively smooth. At point A, the acceleration decreases to $0.20^{{}^{\circ}}$/$s^{2}$, with a jerk of $19.6^{{}^{\circ}}$/$s^{3}$. Without ϵ, the slack angular velocity maintains a high magnitude throughout the motion, resulting in a sharp acceleration peak of up to $2.0^{{}^{\circ}}/s^{2}$ at the initial stage. At the switching point, the slack angular acceleration reaches $0.88^{{}^{\circ}}$/$s^{2}$, with a jerk of $64.7^{{}^{\circ}}$/$s^{3}$. These results indicate that introducing the dynamic gain ϵ effectively smooths the slack motion. However, the parameter selection of *a* and *b* also affects performance. Increasing *a* from 10 to 100 further smooths the slack motion but excessively suppresses its magnitude, thereby reducing the effectiveness of slack-based adjustment. Changing *b* from −100 to −1 enlarges the influence range of ϵ, causing the slack velocity to remain suppressed throughout the motion rather than only near switching points.

#### 5.2.2. Simulation B

**Figure 5:**
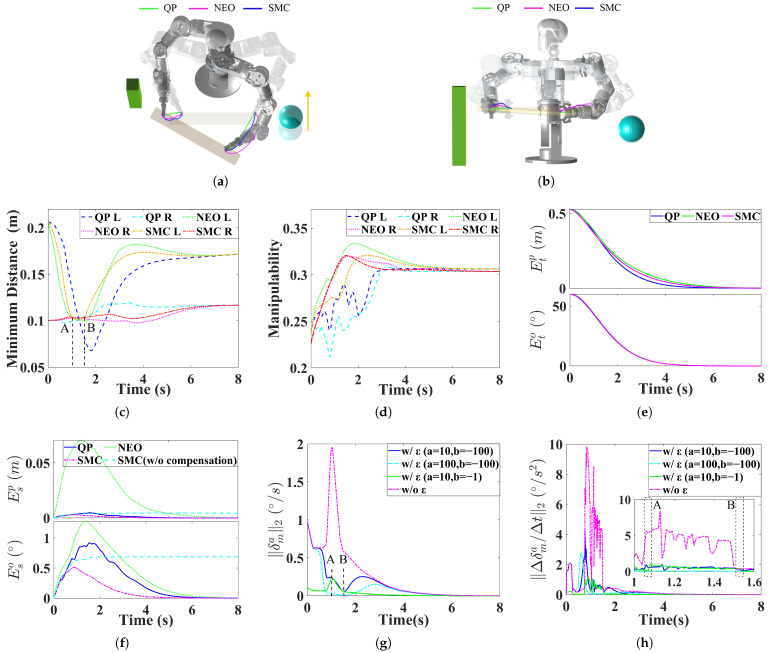
Simulation B. (**a**) Trajectories from a top-down perspective. (**b**) Trajectories from a positive perspective. (**c**) Minimum distance. (**d**) Manipulability. (**e**) Tracking error. (**f**) Synchronization error. (**g**) Slack velocity in SMC. (**h**) Slack acceleration in SMC.

Static and dynamic obstacles are introduced into the scene, as shown in Figure 5a,b. The spherical obstacle moves at a constant velocity along the Y-direction. The proposed SMC is compared with the QP and NEO methods, both equipped with obstacle avoidance capability. The minimum distance to obstacles is used as the criterion for master-slave arm switching.

As shown in Figure 5c,d, the minimum distances to obstacles achieved by the QP, NEO, and SMC methods are 0.068 m, 0.097 m, and 0.100 m, respectively, while the average sum of manipulability of the two arms during motion is 0.58, 0.61, and 0.61, respectively. Compared with QP, SMC with slack velocities increases the performance in both minimum distance and manipulability by 47% and 5.2%, respectively. Moreover, compared with NEO, SMC does not degrade these metrics, indicating that the introduction of slack velocities compensates for the limitations imposed by closed-chain constraints on motion performance.

From Figure 5e,f, similarly to Simulation A, SMC maintains good tracking accuracy and synchronization, whereas NEO fails to satisfy the closed-chain constraint. By introducing the compensation velocities, the maximum synchronization position and orientation errors are significantly reduced from 4.5 mm and 0.69° to 3.0 mm and 0.51°, corresponding to reductions of 33.3% and 26.1%, respectively.

Figure 5g,h illustrate the slack angular velocity and acceleration in the SMC method. It can be observed that, with dynamic gain regulation, the slack angular velocity becomes smoother. At the switching point, the angular acceleration is $0.55^{{}^{\circ}}$/$s^{2}$ with a jerk of $39.1^{{}^{\circ}}$/$s^{3}$, whereas without ϵ, the angular acceleration reaches $5.6^{{}^{\circ}}$/$s^{2}$ with a jerk of $280.0^{{}^{\circ}}$/$s^{3}$.

It should be noted that, in SMC, the joint velocities of the slave arm are still driven toward the gradient direction of manipulability and constrained by obstacle avoidance inequalities. Therefore, the motion of the slave arm continues to improve its manipulability and increase its distance from obstacles. It can be seen from Simulation B that the synchronization constraints do not hinder the improvement of manipulability or the execution of obstacle avoidance tasks for the slave arm.

From the above comparisons, it can be observed that SMC enables synchronized dual-arm motion without trajectory interpolation, while improving manipulability and enabling dynamic obstacle avoidance, all while maintaining high synchronization and tracking accuracy. At the same time, each method exhibits its own advantages and limitations. The SyncTC method achieves highly accurate synchronized tracking of a global trajectory and performs well in terms of tracking and synchronization position errors, but suffers from relatively low manipulability. The QP method can also realize synchronized dual-arm motion without trajectory interpolation; however, due to the absence of slack velocities, it cannot generate end-effector deviations, which limits its capability for dynamic obstacle avoidance of end-effectors. The NEO method improves dynamic obstacle avoidance and manipulability by introducing slack velocities, but lacks closed-chain constraints and thus can only achieve coordinated motion without enforcing closed-chain synchronization.

### 5.3. Experiments

**Figure 6:**
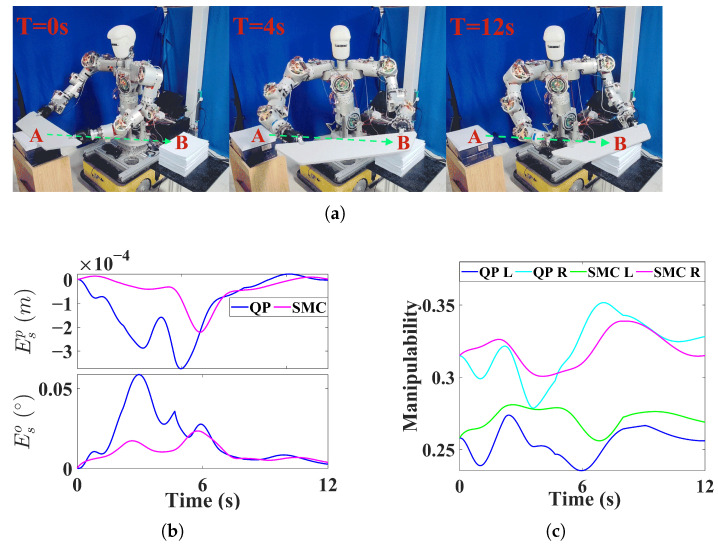
Experiment A. (**a**) The experiment of moving the wooden board. (**b**) Synchronized error. (**c**) Manipulability.

**Figure 7:**
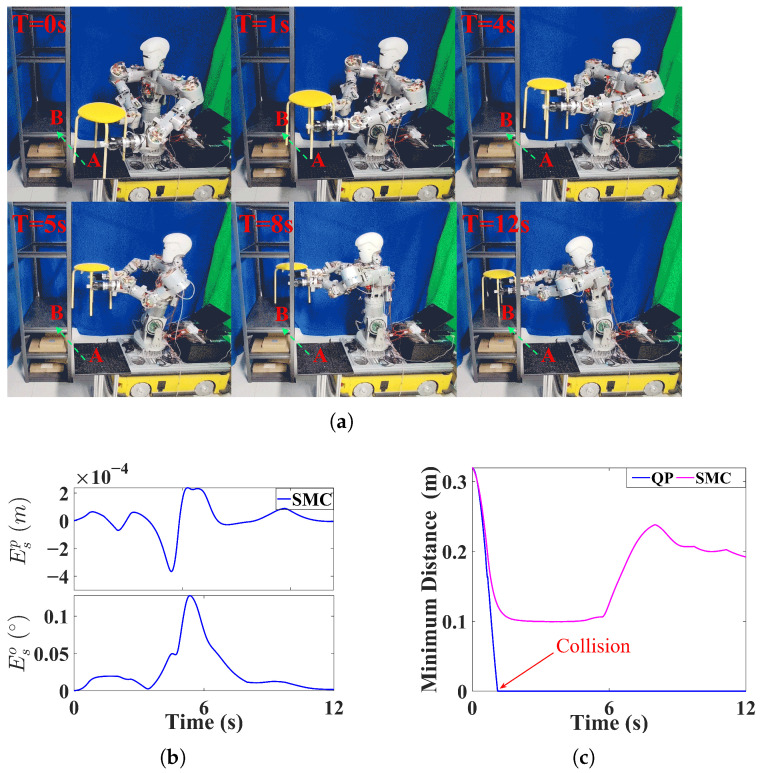
Experiment B. (**a**) The experiment of moving the stool. (**b**) Synchronized error. (**c**) Minimum distance from the obstacle.

Based on the above simulation results, the NEO method cannot guarantee synchronization between dual arms and is therefore unsuitable for transportation tasks involving closed-chain constraints. Consequently, in the experimental validation, we compare the QP-based method with the proposed SMC. We further designed two experimental scenarios, as shown in Figure 6a and Figure 7a. Experiment A involves a dual-arm transportation task in which a wooden board is carried in an obstacle-free environment, while Experiment B involves transporting a stool in the presence of obstacles, requiring avoidance of the side pillars of a shelf. In both experiments, the tasks are specified solely by a target pose, without requiring trajectory interpolation.

As shown in Figure 6b and Figure 7b, SMC achieves high synchronization performance in both experiments. In Experiment A, as shown in Figure 6c, SMC enables the minimum manipulability of dual arms to remain higher than that achieved by the QP method. This is because, in the SMC method, the arm with lower manipulability is selected as the master arm, and its slack velocity is not constrained. As a result, a more balanced manipulability between the two arms can be maintained, preventing either arm from operating at very low manipulability. In Experiment B, as shown in Figure 7c, since the obstacle lies along the straight line between the initial and target poses, the QP method, lacking slack velocity, cannot generate deviation in the end-effector motion and thus fails to bypass the obstacle. In contrast, the SMC method, with slack velocities incorporated, can generate deviation in motion of the end-effectors and successfully avoids the obstacle.

From the experimental comparisons, it can be observed that the slack velocity with synchronization constraints improves both manipulability and dynamic obstacle avoidance in dual-arm synchronized motion. In addition, the selection criterion in the proposed master-slave switching strategy, where the arm with weaker capability for flexible adjustment is assigned as the master arm, maintains balanced performance between the two arms.

## 6. Conclusions

In this paper, we propose a dual-arm reactive synchronized motion controller that enables synchronized motion without trajectory interpolation. By leveraging the spherical geometric velocity constraints, we derive a synchronized desired velocity and introduce a compensation velocity to further reduce the synchronization error. The compensation velocity can significantly reduce the maximum synchronization position and orientation errors by 33.3% and 26.1%, respectively. Furthermore, we refine the slack-velocity constraints in the NEO framework by incorporating velocity-level closed-chain constraints under a master-slave arm strategy. To ensure smooth master-slave switching, dynamic gains are introduced into the weighting matrix. Extensive simulations and experiments demonstrate that SMC maintains synchronized motion while simultaneously improving manipulability and enabling obstacle avoidance. Compared with QP, SMC with slack velocities can increase the performance in both minimum distance and manipulability by 47% and 5.2%, respectively.

In future work, we plan to apply SMC to mobile humanoid robots for dual-arm mobile manipulation and coordination and further consider practical issues such as object compliance, gripper slippage, and state estimation errors. We will also try to explicitly integrate energy consumption into the unified cost function, achieving multi-objective optimization to balance manipulability and energy consumption.

## Figures and Tables

**Table 1:** DH Parameters of humanoid upper-body robot.

Joint	Intial $\theta_{i}$ (°)	$d_{i}$ (mm)	$a_{i}$ (mm)	$\alpha_{i}$ (°)
$\theta_{t1}$	180	−15.5	0	90
$\theta_{t2}$	−90	377	0	90
$\theta_{t3}$	180	34.5	130|−130	90
$\theta_{l1}$|$\theta_{r1}$	90	144.5	0	90
$\theta_{l2}$|$\theta_{r2}$	90	323.5|−323.5	0	90
$\theta_{l3}$|$\theta_{r3}$	90	0	0	90
$\theta_{l4}$|$\theta_{r4}$	90	−266.2	0	90
$\theta_{l5}$|$\theta_{r5}$	90	0	0	90
$\theta_{l6}$|$\theta_{r6}$	90	−248.2	0	90
$\theta_{l7}$|$\theta_{r7}$	180	0	0	90
$\theta_{l8}$|$\theta_{r8}$	0	−250	0	0

## Data Availability

The original contributions presented in this study are included in the article. Further inquiries can be directed to the corresponding author.

## Appendix A

**Proof.** Considering real-world operations in dynamic environments, the system is inevitably subjected to sensor noise, modeling inaccuracies, and external perturbations. Let $d\left(t\right)\in R^{12}$ represent these lumped bounded disturbances, such that $∥d\left(t\right)∥\leq \bar{d}$. Combing the desired velocity defined by the closed-loop law $v={\dot{X}}_{tgt}+k_{p}E={\dot{X}}_{tgt}+k_{p}\cdot \left(X_{tgt}-X\right)$ and the unfolding form $v-\delta =J\left(\theta \right)\dot{\theta }$ of the equality constraint Jx=v, we can obtain

(A1) $${\dot{X}}_{tgt}+k_{p}E-\delta =J\left(\theta \right)\dot{\theta }+d\left(t\right)$$

According to the actual end-effector velocity $\dot{X}=J\left(\theta \right)\dot{\theta }$, the error dynamics of the system is formulated:

(A2) $${\dot{X}}_{tgt}-\dot{X}+k_{p}E-\delta =d\left(t\right)\Rightarrow \dot{E}=-k_{p}E+\delta +d\left(t\right)$$

Furthermore, incorporating the closed-chain constraint $\delta_{s}=M\cdot \delta_{m}$ for the slack velocities of the master and slave end-effectors yields the following error dynamics:

(A3) $$\left[\begin{array}{c} {\dot{E}}_{m}\\ {\dot{E}}_{s} \end{array}\right]=-k_{p}\left[\begin{array}{c} E_{m}\\ E_{s} \end{array}\right]+\left[\begin{array}{c} I_{6\times 6}\\ M \end{array}\right]\delta_{m}+\left[\begin{array}{c} d_{m}\left(t\right)\\ d_{s}\left(t\right) \end{array}\right]$$

where $E_{m}$ and $E_{s}$ are the tracking error vectors of the master and slave end-effectors, respectively. $d_{m}\left(t\right)$ and $d_{s}\left(t\right)$ are the lumped bounded disturbances of the master and slave end-effectors, respectively. Define a following Lyapunov candidate function:

(A4) $$V=\frac{1}{2}E^{T}E$$

Taking its time derivative and substituting (A2) yields:

(A5) $$\dot{V}=E^{T}\left(-k_{p}E+h+d(t)\right)$$

where $h=\left[\begin{array}{c} I_{6\times 6}\\ M \end{array}\right]\delta_{m}$. Applying the Cauchy–Schwarz inequality, we obtain:

(A6) $$\dot{V}\leq -k_{p}{∥E∥}^{2}+∥E∥(∥h∥+\bar{d})$$

To ensure a strict decrease in the Lyapunov function ($\dot{V}<0$), the tracking error must satisfy the following condition:

(A7) $$∥E∥>\frac{∥h∥+\bar{d}}{k_{p}}$$

Since the slack variable $\delta_{m}$ is an optimization variable tightly constrained by the physical limits of the robot joints and the feasible region of the task space, its mapped term h is inherently bounded, implying the existence of a maximum norm ${∥h∥}_{\max}$. Consequently, whenever the error norm exceeds the threshold ${(∥h∥}_{\max}+\bar{d})/k_{p}$, the Lyapunov derivative is strictly negative. This mathematically guarantees that the tracking error E will consistently converge toward, and ultimately remain confined within, the bounded compact set:

(A8) $$\Omega =\left\{E|∥E∥\leq \frac{{∥h∥}_{\max}+\bar{d}}{k_{p}}\right\}$$

Therefore, our proposed SMC guarantees Uniformly Ultimately Bounded (UUB) stability, ensuring reliable tracking performance even under the enforced closed-chain constraints and external perturbations. □

## Appendix B

**Table A1:** A summary table of main symbols.

Symbol	Description
$\theta_{t},\theta_{l},\theta_{r}$	Joint angle vector of the torso, left and right arms.
$\theta ,\dot{\theta }$	Joint angle and velocity vectors of the robot.
$O_{c},O_{l},O_{c}$	Left and right end-effectors, and their center point.
$T_{b}^{l_{sync}},T_{b}^{r_{sync}}$	Homogeneous transformation matrices of the synchronized poses of the left and right end-effectors with respect to $T_{b}^{c_{cur}}$.
$T_{c}^{l},T_{c}^{r}$	Initial relative poses between $O_{c}$ and $O_{l}$, between $O_{c}$ and $O_{r}$.
$T_{b}^{c_{cur}},T_{b}^{l_{cur}},T_{b}^{r_{cur}}$	Homogeneous transformation matrices of the current poses of the left and right end-effectors, and their center point.
$p_{l},p_{r},p_{c}$	Position components in $T_{b}^{l_{sync}}$, $T_{b}^{r_{sync}}$, $T_{b}^{c_{cur}}$.
$p_{m},p_{s}$	Position vectors of the master and slave arms’ end-effectors.
$T_{b}^{l_{init}},T_{b}^{r_{init}}$	Homogeneous transformation matrices of the initial poses of the left and right end-effectors.
$T_{b}^{l_{tgt}},T_{b}^{r_{tgt}},T_{b}^{c_{tgt}}$	Homogeneous transformation matrices of the target poses of the left and right end-effectors and their center point.
J(θ)	Jacobian matrix by stacking kinematic chains.
$v_{l},v_{r}$	Velocity vectors of the left and right end-effectors.
v	Velocity vector of the two end-effectors.
$v_{comp}^{l},v_{comp}^{r}$	Compensation velocity terms for the left and right end-effectors.
$\delta_{l},\delta_{r}$	Slack velocity vectors of the left and right end-effectors.
$\delta_{m},\delta_{s}$	Slack velocity vectors of the master and slave end-effectors.
δ	Slack velocity vector of the two end-effectors.
x	Optimization variable vector.
$J_{m}$	Manipulability gradient.
$k_{p},k_{v},\zeta ,\eta$	Gains.
M	Closed-chain constraint matrix.
$mm_{l},mm_{r}$	Manipulabilities of the left and right arms.
$d_{min}^{l},d_{min}^{r}$	Minimum distances between the obstacles and the left and right arms.
$E_{l},E_{r}$	Tracking error vectors of left and right end-effetors between the current and target poses.
$E_{l}^{p},E_{r}^{p},E_{l}^{o},E_{r}^{o}$	Tracking position and orientation error vectors of left and right end-effetors.
E	Tracking error vector of left and right end-effetors.
$E_{t}^{p},E_{t}^{o}$	Total of dual-arm tracking position, total of dual-arm tracking orientation errors.
$E_{rel}$	Relative pose vector between $T_{b}^{l_{cur}}$ and $T_{b}^{r_{cur}}$.
$E_{s}^{p},E_{s}^{o}$	Synchronization position and orientation errors.
Q,C	Coefficient matrices of quadratic and linear terms in the optimization objective function.
J	Equality constraint matrix.
A,B	Inequality constraint matrix.
$A_{c},B_{c}$	Inequality constraint matrix for the obstacle collision damper.
$\lambda_{\theta }$	Weighted factor corresponding to the joint angular velocity in Q.
$\lambda_{\delta_{l}}^{t},\lambda_{\delta_{l}}^{a},\lambda_{\delta_{r}}^{t},\lambda_{\delta_{r}}^{a}$	Weighted factor corresponding to the slack velocity in Q.
ϵ	Dynamically adjustable gain for slack angular velocity.
a,b	Parameters in ϵ.
Δ	Difference between the manipulabilities of dual arms or between their minimum distances to obstacles.
